# Dysentery, Considered in Relation to the Chemical Changes Occurring in the Different Secretions and Excretions; with Cases

**Published:** 1839-09-07

**Authors:** John D. Griscom


### MEDICAL EXAMINER.

DEVOTED TO MEDICINE, SURGERY, AND THE COLLATERAL SCIENCES.

No. 36.] PHILADELPHIA, SATURDAY, SEPTEMBER 7, 1839. [Vol. II.

DYSENTERY, *considered in relation to the Chemical Changes occurring in the different Secretions and Excretions; with Cases.* By John D. Griscom, M. D.

Notwithstanding the large share of attention that this disease has already received from the medical profession, it is acknowledged that many points of its character are yet imperfectly understood.

While many of the symptoms have been closely observed, and accurately described, others of the most obvious importance are overlooked. The system of inductive reasoning from carefully observed *facts*, that within the last few years has been so happily applied to many departments of medical science, seems never yet to have been brought to bear upon dysentery.

This disease, for which so many discrepant definitions have been given, has recently, with more conciseness than truth, been defined to be "inflammation of the large intestines"*—[Dr. Wm. Stokes.] This definition, although conveying a very inadequate idea of the character of the disease, as far as it goes, will be found sustained by post-mortem examinations, and also by the premonitory symptoms, which are those of inflammatory affections generally. Chilliness, alternating with flushes of heat, nausea, and perhaps vomiting, cephalalgia, anorexia, general soreness, and great prostration of strength, are the usual precursors of dysentery; in from twelve to forty-eight hours these are generally followed by diarrhœa, accompanied by griping and a peculiar twisting sensation about the umbilicus.

The alvine dejections, which are at first thin, copious, and feculent, in a space of time varying from one to four days, or longer, become very small, and are found composed almost entirely of bloody mucus. The desire to evacuate the bowels is now often so constant and violent, that the patient can scarcely be induced to leave the chair, while the accompanying pain undergoes such an exacerbation as to become almost insupportable, being compared by females to the "bearing down pains" of parturition, and several of them have assured me that they were not less severe.

When the tormina and tenesmus are great, the bladder often sympathizes with the rectum, and difficulty of voiding urine adds much to the sufferings of the patient.

These are the ordinary symptoms that accompany acute dysentery in our climate,—to which it not unfrequently happens that many more are added, or many of the anterior symptoms may be wanting, and the characteristics of the disease developed much earlier.

The dejections present many varieties, being often compared by the patients to the scrapings of intestines, or the washings of flesh. After a few days they have not the usual fœtor, but an odour so peculiar, that some think it sufficient to characterize the disease.

Pure blood, in considerable quantities, is often discharged, and lymph so organized as to be mistaken for the mucous coat of the intestines.

Scybala, which are mentioned by many authors, I have seldom seen, and never when they appeared to be of a consistency that could cause irritation; it would seem that they are peculiarly frequent in Great Britain, a circumstance that in some measure accounts for the undue importance attached to them by Cullen and his followers.

The febrile symptoms are exceedingly varied; in some cases they are slight, or entirely wanting, while in others they run very high, and are thought to assume the type of the prevailing fever of the season.

The tongue, which is at first white in the centre, with clean red edges, becomes rough and brown, or dry and chapped over its whole surface,—while in some protracted cases it has been compared to a piece of raw meat.

The skin, the functions of which are badly performed from the commencement, usually becomes harsh and dry in the progress of the disease; and although sometimes moist and clammy, will seldom, if ever, be found to give out its natural healthy secretion.

These, I believe, are the most important symptoms that have ordinarily been observed in acute dysentery; and while they have been dwelt on by all the writers on the subject, from the time of Sydenham to the present, the character of the different secretions and excretions, even of the alimentary canal, have been greatly neglected. It has, indeed, been hinted, that the alvine dejections become acid and irritating, and it appears that some attempt was made to base the treatment on the supposition that they were *acid*; but that any effort was made to test their character, I have been unable to ascertain.

At the suggestion of my friend, Dr. Gerhard, I was induced to undertake the investigation of the chemical changes that occur in some of the secretions and excretions in this disease. For this object, the epidemic at the Philadelphia Hospital, in the summer of 1836, and the subsequent cases occurring in the autumn, offered peculiar advantages.

To Drs. Gerhard and Stewardson I am much indebted for their kindness in affording facilities

* "The first principle that I have to enforce on this subject (and you may take it as an observation based on the soundest pathology) is this, that dysentery is inflammation of the large intestines."

for investigation, which I should otherwise have been unable to obtain.

Moistened litmus paper was found to detect the acidity of the different secretions and excretions very readily, but some difficulty was experienced in obtaining a test of a simple character delicate enough to show their alkalinity; this I, however, accidentally found in the litmus paper, reddened by the insensible perspiration of my own fingers. It was after I had decided on the above tests, and the manner of using them, that an abstract of the paper of M. Donné, "On the Chemical Characters of the Saliva," &c.,* fell into my hands, and I was gratified to find them nearly of the same character as those used by him.

After a number of experiments on various individuals, I was fully prepared to agree with M. Donné, that the saliva in health is always alkaline.

That the perspiration and urine contain a free acid in health, is too well known to require any proof; but, in regard to the alvine dejections, I was more at a stand. After a number of careful observations, however, I have arrived at the conclusion that they are *neutral* in health.

During the first part of the epidemic alluded to, and before I had decided to make it the subject of a thesis, my attention was directed principally to the saliva, which, in severe cases of dysentery, I found to be almost universally acid.

Among the cases that most strongly arrested my attention at this time, was that of a young girl under the care of Dr. Gerhard, who, while in the ward for a different affection, was attacked with dysentery. The saliva, which for several days had been decidedly acid, much to my surprise, I one morning found alkaline. On inquiry, it was ascertained that all the unpleasant symptoms had disappeared about twelve hours previously. Her recovery from the disorder from this time was rapid and perfect.

The results obtained in regard to the saliva were so striking, that I was induced to extend my observations to the cutaneous secretion, urine and alvine dejections, and they have been attended with an interest that more than equalled my expectations.

A comparison of the tests carefully made in about twenty cases of severe dysentery, many of them daily till convalescence, gives the following results:

*First.* The *saliva* was uniformly acid, occasional short intervals excepted, and did not permanently resume its alkaline character till the approach of convalescence.

*Secondly.* The *urine* was generally either *neutral* or *alkaline;* but in uncomplicated cases, resumed its healthy condition sooner than the saliva; in some slight cases it was not changed.

*Thirdly.* The *cutaneous secretion* was generally either *neutral* or *alkaline;* but, like the urine, often retained its healthy character when the cases were not severe.

*Fourthly.* The *alvine dejections*, after the disease was fairly established, generally became *strongly alkaline*, and did not permanently resume their healthy character till the force of the disease was subdued,—and they sometimes became acid before resuming their healthy condition.

In some cases the saliva, perspiration, and urine, would resume their healthy standard, and again suddenly lose it, as if nature, in her struggle with the disease, had gained a temporary advantage which she was unable to retain.

The characters of the alvine dejections were more constant, but sometimes appeared to be acid throughout the disease; I say, *appeared to be acid*, for I recorded them so during the early part of my observations, when they might have been, and probably often were mixed with urine. This is more likely, as I then attached less importance to them than I did subsequently, observing them less rigidly and more seldom.

There can be no doubt that they are often acid at the commencement and close of even very severe cases, for they were sometimes found so when the urine was alkaline; it appeared to me that they were more liable to be acid when they contained fœcal matter.

The following cases, which, for the sake of conciseness, I have thrown into the tabular form, though deficient in many particulars, will, I think, be found to corroborate the statements I have given as far as they go.

* See American Journal of Medical Sciences, No. xxxvii., p. 215

CASE I.—Margaret W——, a large Irishwoman, aged fifty-three, entered the hospital Ninth month 9th, and came under the care of Dr. Gerhard. She was then suffering under a severe attack of dysentery, which commenced about Eighth month 27th, 1837.

| Date, 9th mo'h. | *Alv. Dejections.* General Character, Number, &c. | *Alv. Dejections.* Chemical Character | *Saliva.* | *Perspiration.* | *Urine.* | *Treatment and Diet.* |
|---|---|---|---|---|---|---|
| 10 | 20 or 30 in night; bloody mucus scanty. | No test. | | | | ℞ P. Ipecac. Mass Hydrarg. āā gr. v. Ext. Gentianæ, gr. iv. Ft. Pilul. No. iii. ter in die. P. Jalap. c. ℈ii. at noon. Gum water. Arrowroot. |
| 11 | 12, more copious, feculent, yellowish. | No test. | | | | Pills as yesterday. Tr. Opii ʒss. asan. Enema in evening. Gruel. Gum water. |
| 12 | 6, as yesterday. | Acid?* | Acid. | Neut. | Not tested. | Not changed. |
| 13 | 3, thin, yellow, feculent. | Not tested. | Acid. | Slightly acid. | Alkaline. | Three pills bis in die. Gruel and gum water. |
| 14 | 2, as yesterday. | Not tested. | Slightly acid. | Slightly acid. | Acid. | No change. |
| 15 | As yesterday, more pain. | Acid?* | Slightly acid. | Acid. | Not tested. | Pills as yesterday. P. Jalap. c. ʒiss. |
| 16 | 3, more nat. | Not tested. | Neut. | Acid. | Acid. | Pills as yesterday. |
| 17 | About 10, darker. | Not tested. | Neut. | Acid. | Not tested; passed with pain. | Pills, gruel, flax-seed tea. |
| 18 | None. | | Alkaline. | Acid. | Acid. | Pills bis in die. Cracker and soup. |
| 19 | 2, healthy looking. | Not tested. | Alkaline. | Acid. | Not tested. | Pills bis in die. |

* The reason for doubt here is, that they might have been mixed with urine; experience having taught me that little reliance is to be placed on the patient's statements in regard to it.

CASE II.—Bridget M'L——, a rather delicate-looking Irishwoman, aged 26, entered the hospital on the evening of Ninth month 11th, and came under the care of Dr. Gerhard, labouring under symptoms of acute dysentery of an aggravated character; she was attacked, about the 3d with severe diarrhœa, after the usual premonitory symptoms.

| | *Alv. Dejections.* | | | | | |
|---|---|---|---|---|---|---|
| *Date, 9th mo'h.* | *General Character, Number, &c.* | *Chemical Character* | *Saliva.* | *Perspiration.* | *Urine.* | *Treatment and Diet.* |
| 12 | Several in an hour, thin, yellow, much pain. | Not tested. | Acid. | Slightly acid? | Not acid.* | ℞ P. Ipecac.<br>Mass. Hydrarg. āā gr. v.<br>Ext. Gentianæ, gr. iv.<br>Ft. Pilul. No. iii. ter in die.<br>Pulv. Jalap, comp. ℈ii.<br>Gum water.<br>Arrow-root. |
| 13 | Diminished in number, less pain. | Acid? | Acid. | Alkaline. | Acid. | As yesterday the pills.<br>Sago. |
| 14 | 3, part only painful; examination, some blood. | Not tested. | Slightly acid. | Slightly acid. | Acid. | Pills continued; sago; gum water. |
| 15 | 3, copious mucus, but no blood; slight pain. | Not tested. | Neut. | Slightly acid. | Acid. | Pills twice daily; gruel; gum water. |
| 16 | 3, nearly natural. | Slightly alkaline. | Neut. | Slightly acid. | Acid. | As yesterday. |
| 17 | 6, thin, yellow, with pain.† | Not tested. | Acid. | Acid. | Acid. | As yesterday. |
| 18 | 6, more consistent, dark. | Acid? | Slightly acid. | Acid. | Acid. | As yesterday.<br>Cracker, gruel. |
| 19 | 3, dark, consistent. | Slightly acid? | Slightly acid. | Acid. | Acid. | As yesterday. |
| 20 | 3, natural, no pain. | Acid? | Alkaline. | Acid. | Acid. | No change.<br>Cracker and soup. |
| 25 | 1. | Not tested. | Alkaline. | Acid. | Acid. | Discontinued. |

* Its alkalinity was not suspected.

† Contained some undigested potato, which the patient had eaten in the morning; the change produced in the saliva is remarkable. For reasons before stated, the acidity of the stools in this and the following cases, must be taken with some reservation.

Case III.—Sarah W——, a rather delicate Irishwoman, æt. 25, was admitted into the hospital Ninth month 12, and came under the care of Dr. Stewardson. After being wet, about the 1st inst., she was seized with the usual premonitory symptoms of dysentery, the characteristics of which were soon developed with great severity. On entering the hospital, she was much prostrated, and obliged to be almost constantly on the close stool, when she only passed small quantities of bloody mucus. She was ordered two injections of Tr. Opii, gr. xx. each, with Muc. Lini, and rest.

| 1837 | *Alv. Dejections.* | | | | | |
|---|---|---|---|---|---|---|
| *Date, 9th mo'h.* | *Number, General Characters, &c.* | *Chemical Character* | *Saliva.* | *Perspiration.* | *Urine.* | *Treatment and Diet.* |
| 13 | 4 to 6 per hour; mucus & blood; distressing pain. | Not acid.* | Acid. | Acid. | Not acid. | ℞ Pulv. Ipecac. et opii gr. iii., q. t. h. Muc. Lini, ℥iv. pr. Enema. Muc. Ulmi for drink. Arrowroot. |
| 14 | 1 per hour; same character. | Not tested. | Acid. | Neut. | Acid. | Not changed. |
| 15 | More frequent, with an increase of pain. | Not tested. | Acid. | Slightly acid. | Passed with difficulty. | Same. |
| 16 | Not materially changed, pain very severe. | Not tested. | Acid. | Neut. | Acid. | ℞ Mass. Hydrarg. gr. v. P. Ipecac. gr. vi. Ext. Gentianæ, gr. iv. Ft. Pilul. No. iii. } ter in die. Muc. Ulmi. Sago. |
| 17 | About 6, much pain with last. | Not tested. | Acid. | Neut. | Passed with pain. | ℞ as yesterday. In Pilul. No. iv. divid. 2 q. 3â h. |
| 18 | 8, much pain. | Not tested. | Acid. | Neut. | Alkaline, still painful. | As yesterday, with leeching to anus. Arrowroot. |
| 19 | 2 or 3 an hour, (very numerous) scanty, wholly mucus & blood, much pain. | Not tested. | Acid. | Neut. | Alkaline, painful. | ℞ Plumb. Acet. gr. ii. Pulv. Opii, gr. ii. } q. i. d. P. M. Milk Poult. Muc. Ulmi. Boiled milk. |
| 20 | 2 since midnight; bloody mucous. | Not tested. | Alkaline. | Acid. | Alkaline. | As yesterday. |
| 23 | None. | | Slightly acid. | Acid. | | One pill, 4 t. d. |
| 25 | 1, natural. | Slightly alkaline. | | Acid. | | Pills as yesterday. |

* Alkalinity was not suspected; the probability is, they were alkaline throughout. This patient remained in the ward as assistant, and in a few days all the secretions permanently resumed the healthy standard, with the exception of the saliva, which occasionally gave traces of acidity. This, probably, arose from a chronic irritation of the stomach, under which she appears to be suffering.

Case IV.—John S——, a stout, middling-sized man, aged 52, by trade a coach-maker, born in Philadelphia. After a drunken frolic, was seized, Ninth month 24, with dysentery; stools small, slimy; did not observe blood before entrance. Entered the hospital Ninth month 29th, 1837, and came under the care of Dr. Gerhard.

| 1837 9th mo'h. | *Alv. Dejections.* Number, General Character, &c. | *Alv. Dejections.* Chemical Character | *Saliva.* | *Perspiration.* | *Urine.* | *Treatment and Diet.* |
|---|---|---|---|---|---|---|
| 30 | 8 or 9 since last evening; small, slimy, some pus and blood. | | | | | ℞ P. Jalap. comp. ℈ii. P. Ipecac. gr. vi. Ext. Gent. gr. iv. Mass. Hydr. gr. vi. } t. in d. Com. at 6 P. M. Arrow-root. Gum water. |
| *10th mo.* 1 | Six in last 15 hours; watery, bilious. | Not acid.* | Acid. | | Strongly alkaline. | Same. |
| 3 | Six in 15 hours; no bl'd or slime. | Not seen. | Acid. | Very slightly acid. | Not seen. | Two Pills, 3 t. d. Gruel, gum water. |
| 4 | Bilious, deep yellowish brown | Not acid. | Slightly acid. | Slightly alkaline. | Strongly acid. | One Pill, 3 t. d. Gruel, rice, and gum water. |
| 9 | One, natural. | Neut. | Alkaline. | Acid. | Acid. | Treatment discontinued. |

* Probably alkaline.

Case V.—William G——, a stout Irishman, aged 63, entered the hospital Ninth month 13, and came under the care of Dr. Gerhard. He was attacked with dysentery about the 6th, after a fit of intoxication.

| 1837 9th mo'h. | *Alv. Dejections.* Number, General Character, &c. | *Alv. Dejections.* Chemical Character | *Saliva.* | *Perspiration.* | *Urine.* | *Treatment and Diet.* |
|---|---|---|---|---|---|---|
| 14 | 5 in an hour, yellow, feculent, some mucous. | Acid. | Acid. | Neut. | | ℞ P. Ipecac. gr. vi. Ext. Gent. gr. iv. Mass. Hydrarg. gr. v. P. Pilul. No. iij. } t. d. |
| 15 | More frequent; same character. | Acid. | Very acid. | Neut. | Neut. | Continue pills. Tr. Opii, gr. xx. 3 t. d. by enema. |
| 16 | One, without pain. | Not seen. | Acid. | | | Same. |
| 17 | 2, yellow, more feculent, without pain. | Not seen. | Alkaline. | Neut. | | 2 pills bis in die. |
| 19 | Discharged. | | | | | |
| 27 | Natural. | Very slightly acid. | Alkaline. | Acid. | Strongly acid. | Patient in the out wards, apparently well. |

Case VI.—Jane E——, æt. 62, was admitted into the hospital Tenth month 4th, and came under the care of Dr. Stewardson. Patient is a stout Englishwoman, who was seized, about Ninth month 29th, with a diarrhœa. Stools were at first feculent, but, after about four days, consisted almost wholly of blood and slime.

| 1837 | *Alv. Dejections.* | | | | | |
|---|---|---|---|---|---|---|
| *Date, 10th mo.* | *Number, General Character, &c.* | *Chemical Character* | *Saliva.* | *Perspiration.* | *Urine.* | *Treatment and Diet.* |
| 5 | Numerous, dark, slimy, perhaps blood. | Not tested. | Acid. | Slightly acid. | Not tested. | ℞ Ext. Gent. ʒss.<br>Mass. Hydrarg.<br>P. Ipecac. āā gr. xx.<br>Ft. Pilul. No. xii. } t. i. d.<br>Gruel, tea. |
| 6 | 23, dark, tarry looking. | Alkaline. | Acid. | Acid. | Acid. | As yesterday. |
| 7 | 14, very small. | Strongly alkaline. | Acid. | Very slightly acid. | Very slightly acid. | Pills as before.<br>Tr. Opii, gr. xxv. |
| 8 | 12, previous to last even'g, none since. | Not seen. | Strongly acid. | Acid. | Strongly acid. | Pills as before. |
| 9 | 3 stools, more nat. in app. | Alkaline. | Slightly acid. | Acid. | Acid. | As yesterday. |
| 10 | 12, fœcal, no pain. | Alkaline. | Alkaline. | Acid. | Acid. | No change. |
| 12 | 6, fœcal, slight pain. | Alkaline. | Slightly acid. | Slightly acid. | Acid. | ℞ Acid Nitric. gtt. iv.<br>Aquæ, ℥iv.<br>℥ss. q. 2â h. Pills. |
| 13 | 12, fœcal, consistent, no pain. | Neut. | Slightly acid. | Strongly acid. | Acid. | ℞ Aq. Camph. ℥vi.<br>Acid Nitric. gtt. vi.<br>Tr. Opii, gr. lx.<br>℥ss. q. 2â h. |
| 14 | One, natural, no pain. | No test. | No test. | No test. | No test. | As yesterday. |
| 15 | 2, natural. | Neut. | Slightly acid. | | Acid. | ℞ the same ℥ss. occasionally. |
| 16 | 6, natural. | Slightly acid. | Neut. | Acid. | Acid. | Treatment as yesterday. |

The patient left the ward before the test could be again made.

Case VII.—Robert T——, an Irishman, æt. twenty-six, entered the hospital Tenth month 7th, 1837, P. M., and came under the care of Dr. Gerhard. He is a labourer; has been three years in America; taken while working at the quarries on the Schuylkill, with chills and fevers, about six weeks previous to entrance; has had diarrhœa for two weeks, which two days since became much aggravated, and he then first noticed blood in his stools, which then occurred at intervals of a few minutes. On the evening of entrance he was ordered pulv. jalap. comp. ℈ij. at once, and pulv. ipecac. gr. vi., mass. hydrarg. gr. v., ext. gentian. gr. iv., ter in die.

| 1837 | *Alv. Dejections.* | | | | | |
|---|---|---|---|---|---|---|
| 10*th mo.* | *Number, General Character, &c.* | *Chemical Character* | *Saliva.* | *Perspiration.* | *Urine.* | *Treatment and Diet.* |
| 8 | 30 in the night, pain. | | | | | As on the 7th. Gum water; arrow-root; P. M., Enema, Tr. Opii, gr. xxx. |
| 9 | 20 to 25 in 15 hours, watery, brownish. | Slightly alkaline. | Slightly acid. | Neut. | Strongly acid. | Continue pills. Hop poult. cum Tr. Opii ʒi. to abdomen; Enema cum Tr. Opii gr. xxx. at 7 P. M. |
| 10 | Nearly ev'ry half h'r, covered with lymph. | Strongly alkaline. | Neut. | Neut. | Strongly acid. | ℞ Acid Sulph. dil. gr. iij. Aquæ ℥ii. q. 2â h. Continue pills. Toast, weak tea. |
| 11 | Less than 1 in an hour; yellow; more feculent. | Slightly acid. | Alkaline. | Slightly acid. | | Stop pills. ℞ Acid Sulph. dil. gtt. ii. q. 3â h. Toast, weak tea. |
| 12 | 5 in 24 hours. | Strongly alkaline. | Slightly acid. | Slightly acid. | | Treatment as yesterday. |
| 13 | 7 in 24 hours; more fœcal. | Neut. | | | Slightly acid. | Continue acid and diet. Tr. Opii gtt. x. t. in d. pr. Enema. |
| 14 | 4, more consistent. | Alkaline. | Slightly acid. | Slightly acid. | Alkaline. | Continue acid, and mutton at dinner. |
| 15 | None for 36 hours. | | | | | Ol. Ricini, ʒii. |
| 16 | 13, some pain. | | | | | Continue acid, full diet. |
| 19 | About 1 a day, since 16th. | Slightly acid. | Slightly acid. | Acid. | Alkaline. | Treatment for dysentery suspended. |

This patient, after the dysentery had ceased, was seized again with intermittent fever, which may have been the cause that prevented the secretions from fully resuming their healthy state.

Case VIII.—John G———, æt. forty-four, entered the hospital Tenth month 14th, 1837, and came under the care of Dr. Stewardson. He is a large, stout man, of intemperate habits. He was attacked with dysentery on the evening of the 10th, after wandering about the country several days in search of work, and subsisting chiefly, and, indeed, according to his account, almost solely on fruit and water. His stools were at first feculent; but on the 14th blood appeared there, whose consistence being "slime, blood, and froth." On entering the hospital, he was much prostrated; had a small, cardial pulse, very anxious countenance, confused intellect.

| 1837 | *Alv. Dejections.* | | | | | |
|---|---|---|---|---|---|---|
| 10*th mo.* | *Number, General Character, &c.* | *Chemical Character* | *Saliva.* | *Perspiration.* | *Urine.* | *Treatment and Diet.* |
| 15 | One about every 5 minutes; slimy and bloody; severe pain near anus. | Strongly alkaline. | Slightly alkaline. | Acid. | Alkaline. | V. S. ℥xij.—Tr. Opii ʒi. cum Muc. Lini, ℥ii. pr. Enema. ℞ Ac. Nitrici, gtt. vi. Tr. Opii gtt. lx. Aq. Camph. ℥vi. ℥ss. q.h. Hop poultice to abdomen. Gruel and gum water. P. M., leeches xxv to anus. Continue poultice. |
| 16 | 12 since 10 o'clock last evening; same consistence. | Alkaline. | Slightly alkaline. | Acid. | Strongly alkaline. | ℞ the mixture as yesterday. |
| 17 | 30 in 24 hours; small; no increase of pain; yellowish. | Alkaline. | Neut. | Neut. | Alkaline. | Continue mixture; Muc. Ulmi, and poultices. |
| 18 | 7 or 8 yesterday, 2 to-day; no pain; not seen. | | Very slightly acid. | Neut. | Alkaline. | Mixture and Muc. Ulmi. |
| 19 | 3, more consistent; without blood or slime. | Slightly acid. | Neut. | Slightly alkaline. | Alkaline. | Mixture ℥ss. q. 4â h. Muc. Ulmi; Arrow-root. |
| 20 | None. | | Alkaline. | Acid. | Alkaline. | Mixture ℥ss. q. 4â h. Muc. Ulmi; Mutton Soup. |
| 24 | | | | | | Medicine discontinued. Muc. Ulmi; Mutton Chop. |
| 25 | 3 in 24 hours; no pain. | Slightly acid. | Slightly acid. | Acid. | Acid. | |
| 31 | 2 in 24 hours; no pain. | Not tested. | Not tested. | | | |

*Causes.*—Among the supposed causes of dysentery, are usually enumerated sudden vicissitudes of atmospheric temperature from heat to cold, functional derangement of the liver, malaria, bad food, irregular habits of living, excessive fatigue, accompanied by poor diet, scybala, or retained fæces, depressing moral influences, and, by some authors, contagion.

That sudden transitions from heat to cold have a powerful influence in producing dysentery, is now strongly established. The disease appears most frequently and with the greatest violence at the seasons of the year when these changes are greatest,—in our autumn, when warm days are succeeded by cool nights, the lower classes, who are exposed to these changes, are very liable to be attacked with dysentery. Its ravages among sailors going South, and particularly those engaged in the East India trade, are well known.

Whether disease of the liver, either functional or organic, should ever be considered a cause of dysentery, admits certainly of strong doubts. Highly respectable testimony from the different parts of Europe, and even from the East Indies, where the disease puts on its worst forms, seems to discountenance the idea altogether.

Dr. Ballengall, as quoted by Dr. Mackintosh, found not the smallest trace of disease in the structure of the liver. Dr. Twining, in his valuable work on the diseases of Bengal, says:—"The assumption of functional disorder of the liver as the ordinary cause of dysentery, rests on a very equivocal foundation, and must be admitted with great caution." Dr. Cheyn found the liver sound in a majority of cases, though often otherwise.

Dr. J. Johnson says, in numerous fatal cases of dysentery in hot climates, no structural derangement of the liver can be observed.

In relation to the influence of malaria, I am not prepared to speak.

Few persons now believe that scybala have any thing to do in the production of the disease.

To the influence of depressing moral causes I am disposed to attach considerable importance: in a great majority of the cases, I have noted the patients were suffering under an influence of this character.

A considerable proportion had been exposed to wet after being heated, or had slept out all night after a drunken frolic,—and a number had suffered from excessive fatigue, and want of proper food.

There is certainly much evidence in favour of the contagiousness of certain forms of dysentery; although my own observations would not warrant a positive opinion, I am strongly inclined to believe that it does exist.

*Pathology.*—The careful dissections recently made in various parts of the world, show that the force of the disease falls early upon the mucous coat of the great intestine, which uniformly gives evidence of inflammation, even when an independent affection has cut the patient off in the early stages. In cases of death from the disease, the most extensive ulceration and destruction are often found, not only of the mucous, but also of the cellular, muscular, and even peritoneal coats. Evidence of the latter circumstance is often found in adhesions between the folds of the intestines, or the intestines and omentum; or, in less fortunate cases, death suddenly supervenes, and the contents of the intestine are found effused into the cavity of the abdomen.

To show the extent to which these lesions may occur, without producing death, as well as to illustrate the severe character of the disease as it occurs in Bengal, the following extract from Dr. Twining's work, already quoted, will be found interesting:

"The ulcerations within the great intestines, are generally most numerous and most extensive at the cœcum and first portions of the colon: the valvula ileo-colica has in some cases been quite destroyed by ulceration, and the lower end of the ileum has formed an intussеption in the cœcum, and, becoming there strangulated, has caused death. In a few more fortunate instances of intussеption, when the lower portion of the ileum descends into the cœcum, and forms a circumscribed tumour in that region, attended with suppression of stools and rapid pulse, which prove the obstruction of the canal of that part, the strangulated portion sloughs off, after adhesive union, to the adjacent parts, has taken place, so as to maintain the entire continuity of the canal; and then the stools are again passed together with masses of slough, or entire portions of the intestinal tube, and the patient slowly recovers. In eight years I can mention five cases of this sort, two of which have recovered."

In several dissections that I have seen, an enormous fungous thickening of the coats of the large intestine, was found.

The small intestines are said to be generally healthy; I have often seen them implicated, though in a much less degree than the large,—in most cases the mucous coat of the lower portion only being reddened and softened.

The liver, in all the cases that I have seen, gave no positive evidence of disease,—nor did there, in a large proportion of the cases, appear to be any deficiency of its secretion during life.

The stomach, in a considerable proportion of instances, is certainly implicated. The early nausea, and subsequent anorexia, in conjunction with the post-mortem appearances, strengthen this conclusion. In several cases I have seen the mucous coat thinned and softened, or thickened and mammillated.

Dr. Cheyn, in his account of dissections at the Whitewash Hospital, Dublin, says:—"The mucous membrane of the stomach, and small intestines, sometimes present an inflamed appearance."

Dr. Abercrombie testifies to the same facts.*

The altered state of secretions and excretions shows extensive functional derangement of the organs of assimilation and excretion, particularly of the alimentary canal and skin. Recamier, as quoted by Dr. Eberle, states:—"When death takes place early and suddenly in dysentery," we find "only *acrid fluids, which are sometimes so irritating as to cause erysipelas in the parts with which they come in contact.*"—Revue Medicale, Jan., 1825, p. 23.

There appears to me strong grounds for the supposition that this derangement depends, at least in part, upon a shock received by the nervous system. The influence of moral causes in the production of this disease, and the effects of dissipation, certainly give strength to this view. The severe pains in different parts of the abdominal cavity, and the close sympathy between the various viscera supplied by the sympathetic nerve, as the stomach, liver, kidneys, and bladder, go far to convince me that the functions of this nerve are deeply implicated.

And do not the effects produced by the powerful narcotics and anti-spasmodics, as opium, nux vomica, camphor, and tobacco, give additional weight to the supposition?

In regard to the liver, I have little to add to what has already been said under the head of symptoms. I do not consider it improbable that functional derangement of this viscus may occasionally act as a predisposing cause to the affection.

*Prognosis.*—Sporadic cases of dysentery, as they occur in our climate, almost always terminate favourably. But, during the epidemics of summer and autumn, when the disease assumes

* See Pathological and Practical Researches on the Diseases of the Stomach, p. 226.

all the violence that characterizes its course in tropical regions, the results are widely different, as many cases are then found to resist the most judicious management.

The state of the stools, in connexion with the tormina and tenesmus, have hitherto been most relied on as grounds of prognosis in the early stages; as the disease progresses, the patient's strength, and the whole train of intercurrent symptoms, are to be taken into account; but, in every stage, the state of the moral feelings should be closely attended to.*

Should the stools become larger and more feculent while a diminution of pain takes place, the patient may be considered improving; and if, at the same time, a part or all of the secretions be found approaching the healthy standard, or losing their vitiated character, while no peculiar moral depression exists, still greater hopes may be indulged.

But, on the contrary, should the stools continue small, slimy, and streaked with blood, the pain undiminished, and the secretions and excretions retain their abnormal character, we may be sure the force of the disease is unsubdued. If the stools become dark, and have a very offensive, cadaverous odour, while prostration continues, even though there be a complete cessation of pain, and the patient thinks himself nearly well, (as he often will,) we may fear a speedy and fatal termination, as mortification has probably ensued.

An unfavourable prognosis should, however, be given with great caution; for there are few diseases where recoveries have taken place where there seemed so little reason for hope, (witness the instance recorded by Dr. Twining, as given under the head of pathology.)

Discharges of pure blood, though calculated to alarm the patient and friends, are generally followed by an alleviation of symptoms, and in the early stages particularly should be looked upon as favourable.

*Treatment.*—I shall not attempt to go into an enumeration of all the remedies that have been lauded for their supposed efficacy in dysentery, but endeavour to confine myself to the indications deduced from the pathology; believing that if these are consistently met with sound reason, we shall have no occasion for resorting to the empirical practice, which it is acknowledged has too much obtained in the treatment of this formidable affection.

The primary indications thus deduced appear to me to be—

*First.* To arrest inflammation.

*Secondly.* To remove the irritation of the alimentary canal, and alleviate pain; and,

*Thirdly.* To restore the healthy character of the secretions.

In regard to the first indication, little need be said; the usual antiphlogistic treatment should, of course, be resorted to. Topical bloodletting will be often found highly beneficial. In the early stages, when the tormina are very severe, leeching to the anus often produces the most decided relief. This, indeed, might be naturally inferred from the fact that spontaneous discharges of blood, under these circumstances, are always followed by an alleviation of symptoms.

With the second indication, is involved the theory of the nature of the secretions and excretions, and the affection of the nervous system. From what I have seen, I am abundantly convinced that the course of the disease may be greatly shortened, and the amount of suffering much diminished by an attention to these points.

It has long been inferred from the rational symptoms, that the alvine dejections are acid and irritating; and demulcent medicines have been used with a view of counteracting these effects, with undoubted benefit.

We may certainly infer that a knowledge of the chemical characters upon which these irritating qualities depend, will enable us to counteract them with greater certainty, and much more effectually.

Nor does this conclusion rest upon mere supposition. It may, I think, be safely inferred from the foregoing cases, that the alvine dejections are, as a general rule, *strongly alkaline.* Now, it is a well known fact, that the inhabitants of the West Indies have been taught by experience, independent of, and, perhaps, at that time, in the face of all theory, that acid fruits are frequently the proper diet for dysenteric patients, and under which alone, without any other medicine, they will often rapidly recover.

Prof. Chapman* gives the case of a lady, who, after having received the best medical advice of Europe without avail, was cured of a chronic diarrhœa, by living entirely on oranges.

Dr. S. G. Morton, in his last edition of Mackintosh's Practice, says of a mixture of nitric acid, camphor, water, and laudanum,† that he has cured more dysenteric patients with it "than with all the other internal remedies collectively;" and from what I have seen of its effects, I should not think its virtues overrated. In the case of John G——, one of the most threatening I have ever witnessed, its effects were certainly remarkable. In case sixth, and several that I have not reported, the most marked benefit followed its administration. Although I consider the acid an important agent in neutralizing the acid character of the contents of the alimentary canal, and giving tone to the system, it may be inferred from my notions of the pathology that I attach no small degree of importance to the anodyne influence of the camphor and laudanum.

In the treatment of case seventh, Dr. Gerhard

* Since writing the above, Dr. Gerhard has informed me that the fatal termination of a case under his care, could be accounted for on no other ground than the severe moral depression under which he was labouring.

* See Remarks on Chronic Fluxes of Bowels, by N. Chapman, M. D., in American Journal of Medical Sciences, No. xxvii., p. 97.

† Perhaps essentially the same with Hope's mixture. See the prescription for G——, case eighth.

procured most decided benefit by the exhibition of sulphuric acid alone.

My preceptor and highly esteemed friend, Dr. Noble, has informed me that he cured cases that resisted all other treatment, by the exhibition of chloride of sodium, dissolved in common vinegar.

Marked improvement followed the administration of diluted nitric acid alone, in case sixth,—which it was, however, thought best to change for Dr. Morton's prescription, as represented in the table.

After, or in connection with, gentle purges, the neutralizing medicines and anodynes, the different mucilages are very proper as diluents; besides which, Dr. Abercrombie recommends "equal and gentle pressure of the abdomen by a roller of elastic flannel, a remedy which has been strongly recommended as of much efficacy in all the stages and forms of dysentery."

Warm hop cataplasms applied to the abdomen, will often be found to produce great relief.

For the last indication, the remedies have usually been selected from those supposed to stimulate the secretions.

The preparations of mercury, and particularly calomel, have long been favourites. Of the latter article, on the vague supposition that the liver was somehow or other involved, enormous quantities have been administered,—it is to be feared, too often with any thing but a salutary effect.

Dr. Annesley, whose experience in this disease will not be questioned, thinks the constitutional effects of mercury rather accelerate, than arrest the unfavourable termination.

Dover's powder has a deserved reputation; and of all the remedies that I have seen used for this indication, the combinations of ipecacuanha have been attended with the most satisfactory results. Dr. Twining says:—"A great variety of trials have satisfied me that the praise formerly bestowed on large doses of ipecacuanha, as an anti-dysenteric, are in many instances founded on the most just grounds, and the remedy appears advisable in the early stages of almost all ordinary cases of acute dysentery." *

The following is his formula:

℞. Pulv. Ipecac. gr. vi.
Ext. Gentianæ gr. iv.
Mass. Hydrarg. gr. v.
M. ft. pilul. No. iij.

The whole to be taken three times daily.

He says:—"The extract of gentian, as above described, almost always restrains the emetic properties of the ipecacuanha, but does not interfere with its anti-dysenteric effect." "The efficacy of the two remedies above named is much increased by the addition of blue pill." † This prescription has been extensively used at the Almshouse Hospital; and, as far as I have been able to judge, with highly gratifying results. In case fifth, where, from the uniform acidity of the stools, the acid treatment was not indicated, the relief it produced was very striking and effectual. The results in cases first, second, and fourth, besides a number of others not reported, were scarcely less satisfactory.

Much of the success in the treatment of this disease, will depend upon a careful attention to the diet. In the early stages, very small quantities of such articles as are digested in the upper portion of the alimentary canal, should only be allowed; for this purpose, the farinaceous articles generally, and mucilaginous drinks, will be found well adapted. Should the case become protracted, and the patient's strength much reduced, such articles as chicken broth, beef essence, or even the diffusible stimulants, must often be resorted to.

* Diseases of Bengal, 2d ed., vol. i., pp. 93 and 94.

† Diseases of Bengal, 1st ed., p. 38.

